# Key underwater habitat types of the northern Baltic Sea: An assessment of biodiversity and ecosystem functions

**DOI:** 10.1007/s13280-026-02391-1

**Published:** 2026-05-14

**Authors:** Laura Kaikkonen, Susanna Jernberg, Fiia Haavisto, Christoffer Boström, Camilla Gustafsson, Suvi Kiviluoto, Kirsi Kostamo, Sanna Kuningas, Harri Kuosa, Essi Lakso, Annica Långnabba, Henrik Nygård, Mats Westerbom

**Affiliations:** 1 Finnish Environment Institute Syke, Latokartanonkaari 11, 00790 Helsinki, Finland; 2 University of Helsinki, Helsinki, Finland; 3 Metsähallitus, Parks and Wildlife Finland, Opastinsilta 12 C, 00521 Helsinki, Finland; 4 Environmental and Marine Biology, Åbo Akademi University, Henriksgatan 2, 20500 Turku, Finland; 5 Tvärminne Zoological Station, University of Helsinki, J. A. Palméns Väg 260, 10900 Hanko, Finland; 6 Natural Resources Institute Finland (Luke), Latokartanonkaari 9, 00790 Helsinki, Finland; 7 Natural Resources Institute Finland (Luke), Wolffintie 35, 65200 Vaasa, Finland

**Keywords:** Baltic Sea, Benthic habitats, Biodiversity, Ecosystem functions, Expert knowledge, Pelagic habitats

## Abstract

Effective management of marine ecosystems requires understanding linkages between biodiversity and ecosystem functioning. While key species have been identified in many marine systems, the functions provided by different habitats have not been comprehensively synthesised in the region, limiting the ability to identify key habitats. Here, we assess the current state of knowledge on biodiversity and ecosystem functions associated with benthic and pelagic habitat types in the northern Baltic Sea. Drawing on literature, data and expert assessments, we evaluated 49 habitat types, identifying 750 habitat type-function linkages. Of these, 35% were supported by Baltic Sea literature and 29% by expert opinion, while one-third remained unassessed due to data gaps. Our findings highlight key habitat types with high functional importance and reveal major knowledge gaps and research biases across habitats. This synthesis provides a science-based foundation for marine spatial planning, ecosystem service assessment, and sustainable management of northern Baltic Sea biodiversity.

**Supplementary Information:**

The online version contains supplementary material available at https://doi.org/10.1007/s13280-026-02391-1.

## Introduction

Marine ecosystems are under multiple human threats, causing loss of essential functions and services (Keck et al. 2025). To effectively target conservation and restoration efforts, it is essential to understand the linkages between marine biodiversity and ecosystem functioning (Strong et al. 2015). While key species providing important ecosystem functions have been identified in many marine systems (Snoeijs-Leijonmalm and Andrén 2017; Wu et al. 2019; Wernberg et al. 2024), we still lack comprehensive syntheses of the overall functions provided by underwater habitat types characterized by broader species assemblages in many regional seas, including the Baltic Sea.

Ecosystem functions are defined as physical, chemical and biological processes that transform and translocate energy or materials in an ecosystem (Naeem et al. 1994; Paterson et al. 2012). Therefore, ecosystem functions underpin several ecosystem goods and services (Armoškaitė et al. 2020; Jernberg et al. 2024). Functions denote processes that sustain ecosystem dynamics, encompassing mechanisms maintaining biodiversity and ecosystem stability. While global and regional syntheses have demonstrated that certain marine habitats, such as seagrass meadows, kelp forests, and saltmarshes, play disproportionate roles in nutrient cycling, carbon storage, and biodiversity support when measured per unit area (Barbier et al. 2011; Salomidi et al. 2012), comparable synthesis encompassing a range of underwater habitat types and the functions they provide are still limited. This gap constrains our ability to evaluate their relative functional importance and to translate habitat assessments into ecosystem service and management frameworks (but see e.g., Armoškaitė et al. 2020 for linking ecosystem functions to habitat and services in the Baltic Sea).

Understanding linkages between habitat types and ecosystem functions is essential for spatial management and conservation actions, including restoration, to identify priority areas for diverse functions. As a result, ensuring functioning of marine ecosystems is addressed in several marine policies, including the European Marine Strategy Framework Directive (MSFD, 2008/56/EU), and through commitments to retain important ecosystem components (e.g., Water Framework Directive, 2000/60/EC, and Habitats Directive, 1992/43/EEC). The Convention on Biological Diversity (CBD) further calls for conserving and preserving biodiversity and ecosystem functioning in all ecosystems, including marine systems. Particularly the related Kunming-Montreal Global Biodiversity Framework (GBF) (CBD 2022) sets ambitious targets for 2030 to ensure that “by 2050, biodiversity is valued, conserved, restored and wisely used, maintaining ecosystem services”. The EU Nature Restoration Regulation (EU 2024/1991) further aims to enhance ecosystem functioning through measures, such as restoring seagrass beds. Broad-scale evaluation of ecosystem functionality across habitats is therefore critical to meet these policy commitments.

To be able to consider ecosystem functioning in management actions, it is essential to identify the abiotic and biotic components maintaining key functions. Different habitat types provide different functions, this being a result of differing species assemblages and abiotic factors that create spatial and temporal variation in ecosystem functions in natural populations (Alsterberg et al. 2017; Hector and Bagchi 2007; Reiss et al. 2009).

Here, we present an assessment of the biodiversity and ecosystem functions provided by benthic and pelagic habitat types of the northern Baltic Sea based on a classification of habitats in Finland and the EU. The Baltic Sea is used as a case study in this assessment due to its rich research history (Reusch et al. 2018) and available data from a range of habitat types. Previous work on ecosystem functions in the region has focused on biogeochemical functioning (Kuliński et al. 2022) and pelagic and benthic processes, such as nutrient cycling, primary production, and species-specific interactions (e.g., Ask et al. 2016; Gustafsson et al. 2017). Yet, as previous studies have largely focused on specific ecosystem components such as species and a limited number of habitats (but see e.g., Gustafsson and Boström (2014)), we have an incomplete understanding of how different habitat types contribute to various ecosystem functions. Understanding these processes is essential for assessing the resilience of marine ecosystems, especially in the face of increasing anthropogenic pressures, such as eutrophication, climate change, and habitat degradation (Boström et al. 2024). This information is crucially needed to make informed decisions about management measures to improve the environmental status of the Baltic Sea as well as identifying areas of high ecological value that contribute to ecosystem services (Inacio et al. 2020; Jernberg et al. 2024).

The aim of this study is to determine key habitat types that have particularly high contributions in maintaining biodiversity and ecosystem functions along the Finnish coast of the northern Baltic Sea. We use literature review and expert assessments to link habitat types to selected functions and estimate the relative contribution of the habitat types sustaining these functions. In doing so, this review aims to support sustainable management by providing updated science-based information on biodiversity and ecosystem functionality to spatial planning processes.

## Materials and methods

### Study area

This study focuses on the northern Baltic Sea, characterized by brackish water with a salinity declining from 7 in the northern Baltic Proper through the Bothnian Sea to 2 in the northern Bothnian Bay (Kautsky and Kautsky 2000) and eastern Gulf of Finland. Within this unique setting, diverse habitat types support biodiversity and provide vital services to marine life and humans. The number of species in the area is low compared to fully marine ecosystems, and the area is occupied by a mixture of species of marine, brackish and freshwater origin (Ojaveer et al. 2010). With a population of over 85 million people in its catchment area, the Baltic Sea is one of the most human-impacted sea areas in the world, and human activities in the region continue to increase (Reckermann et al. 2022). Land-based runoff leading to eutrophication is the most severe problem in the Baltic Sea with nutrient inputs increasing primary production and causing cyanobacterial blooms, which increase sedimentation and oxygen deficiency in coastal bottom waters (Carstensen et al. 2014). While long-term efforts to reduce nutrient inputs are starting to show as improvement in eutrophication state (Andersen et al. 2017), large areas of the Baltic Sea seafloor still suffer from hypoxic and anoxic conditions (Virtanen et al. 2019). Low species numbers together with the physiological stress caused by harsh winter conditions, low water salinity and eutrophication make the system vulnerable to additional stress caused by humans, such as climate change (Reckermann et al. 2022). As a result, many of the Baltic Sea ecosystem components are in a poor environmental state (HELCOM 2023).

### Habitat types and habitat complexes

There are several habitat classification systems for describing underwater marine habitats in the Baltic Sea, including those applied under the Marine Strategy Framework Directive and the Habitats Directive. In this study, we use the marine habitat type definitions in national threatened status assessment (Kotilainen et al. 2018b) based on the Finnish Red List of Ecosystems (RLE) classification (Kontula and Raunio 2019) as it provides a management-relevant and ecologically meaningful framework for linking habitats to ecosystem functions. By defining habitat types based on dominant biota, the RLE captures the biological and structural features that underpin ecosystem processes, while offering greater detail than other habitat classifications. This makes it particularly suitable for assessing how distinct habitat types contribute to ecosystem functioning and for supporting conservation and management decisions. RLEs are in line with the pan-European EUNIS habitat classification system and the HELCOM Underwater biotope and habitat classification system (HELCOM HUB) used in the Baltic Sea (HELCOM 2013) .

Our assessment comprises 40 benthic and 9 pelagic habitat types. RLE defines habitat type as a distinct land or water area where similar environmental characteristics and biota exist, and which differ from other habitat types based on these characteristics. The habitat types are divided into eight larger groups based on dominating biota (such as perennial algae or invertebrates) and these groups are further divided into habitat types characterized by a specific species or species group (such as hard benthic habitats characterized by *Fucus vesiculosus*). Some of the habitat types are defined by habitat-forming species (e.g. *Zostera marina* meadows, *Fucus* beds), while others are characterized by the presence of particular species (e.g. *Macoma balthica* dominated habitats), which serve as indicators within this framework. In addition to the original list of underwater biotopes (Kotilainen et al. 2018b), we included soft benthic habitats characterized by *Phragmites australis* in our review, as it is an increasingly common habitat type in the littoral zone (Altartouri et al. 2014).

Most of the functions were assessed for individual habitat types, but the assessment of fish habitat function (fish spawning, nursery grounds and adult feeding areas) was conducted using habitat type groups instead of habitat types characterized by a specific species, as many coastal fish species rely on a mosaic of structurally diverse habitats during different life stages. In addition, we included five larger habitat complexes defined in the EUNIS based on the EU habitats directive Annex I habitats (Moss 2008) (including flads- and glo-lakes, estuaries, reefs and sandy banks) as these and the habitat type groups provide physically favourable conditions for different life stages of fish. We assessed the importance of the habitat type groups and habitat complexes to nine fish species (herring *Clupea harengus membras*, sprat *Sprattus sprattus*, smelt *Osmerus eperlanus*, perch *Perca fluviatilis*, pikeperch *Sander lucioperca*, whitefish *Coregonus lavaretus*, salmon *Salmo salar*, trout *Salmo trutta* and pike *Esox lucius*) that have economic importance for Finnish commercial fishery and one fish species (grayling *Thymallus thymallus*) that has been assessed as critically endangered in the Baltic Sea.

### Ecosystem and biodiversity functions

We included 10 ecosystem functions and six biodiversity and habitat functions (hereafter ‘biodiversity functions’) in our review (descriptions in Tables 1, 2). Ecosystem functions are based on Giller et al. (2004) and Strong et al. (2015) with some modifications and include functions, such as primary production and bioturbation. Biodiversity functions describe attributes contributing to overall biodiversity including maintenance of threatened species and genetic variation.

**Table 1:** Summary and descriptions of the assessed ecosystem functions. Ecosystem functions are divided into larger ecosystem process categories (1–5) following the examples presented by Giller et al. (2004) and Strong et al. (2015) with some modifications

Ecosystem function	Description
Net primary production	Primary production is the rate at which energy is converted to biomass via photosynthesis or chemosynthesis by marine primary producers such as vascular plants, macroalgae, phytoplankton and chemosynthetic microbes. In temperate marine ecosystems, vascular plants, macroalgae, phytoplankton and chemosynthetic microbes account for most primary production thereby providing energy and nutrients to higher trophic levels in the food webs (Strong et al. 2015)
Secondary production	In secondary production, heterotrophs such as heterotrophic microbes, zooplankton, benthic invertebrates and fish incorporate organic matter containing chemical energy to their biomass. Secondary production supports higher consumers and is a usable indicator of trophic capacity, health and functioning of aquatic ecosystems (Rodil et al. 2020)
Net oxygen production	Oxygen production happens as a by-product of photosynthesis of aquatic primary producers, forming a main source for water column oxygenation in coastal areas. Oxygen is essential for aerobic life and affects biogeochemical cycling of carbon, nitrogen and phosphorus among other elements. For instance, oxygen released from the roots of aquatic plants enhances formation of oxidized zones in sediments favourable to denitrification that results in nitrogen removal from the system (Caraco et al. 2006)
Organic matter decomposition and biodeposition	Organic matter decomposition refers to degradation and breaking down of organic matter through direct metabolism of benthic organisms or by enhancing microbial activity. Degradation and burial of organic matter are of importance for both cycling and deposition of carbon and nutrients (Arndt et al. 2013). Biodeposition is deposition of organic matter processed by filter feeders, usually in the forms of faeces and pseudofaeces
Nitrogen and phosphorus retention and removal	Nitrogen and phosphorus are retained in coastal zones through uptake by vegetation and particles, slowing their flow to the sea (Asmala et al. 2017, 2018). Long-term removal occurs mainly via denitrification and phosphorus burial (Carstensen et al. 2020). Aquatic plants support these processes by storing nutrients, creating oxic-anoxic interfaces, and hosting denitrifying microbes (Soana et al. 2020). Benthic macrofauna further enhance nutrient removal by improving sediment oxygenation and biogeochemical cycling
Carbon retention and removal	Marine sediments store significant amounts of carbon, termed “blue carbon”, strongly mediated through macrophytes like seagrasses and perennial algae (Wikström et al. 2025). These plants sequester CO_2_ in biomass and sediments but also release carbon via root exudates. Perennial algae on rocky shores contribute standing biomass, and some macroalgal production is exported and buried in sediments or the deep ocean, aiding long-term carbon sequestration (Buck-Wiese et al. 2023)
Water filtration by filter feeders	Benthic and pelagic suspension feeders play an important role in purifying water through physical retention of suspended particles through removing particles from the water by their feeding behaviour and by promoting their sedimentation. This natural filtration helps maintain healthy marine environments, supports biodiversity, and reduces the impact of harmful algal blooms and eutrophication
Seabed stalibization	Seabed stabilization involves reduced sediment resuspension, nutrient binding processes, and enhanced sedimentation and trapping of suspended solids from the water column, decreased water turbidity and improved water quality. Both physical properties of the sediment and biotic factors e.g. macrofauna and vegetation affect sediment erodibility and resuspension with submerged vegetation, such as seagrasses playing a key role by attenuating waves, reducing currents, and trapping suspended solids and nutrients (Meysick et al. 2019)
Bioturbation and bioirrigation	Benthic macro- and meiofauna modify the sediment primarily through bioturbation (reworking of particles) and bioirrigation (solute transport in the sediment). Both processes affect the sediment biogeochemistry and affect other ecosystem functions such as organic matter mineralization, nutrient regeneration and trace element cycling

**Table 2:** Summary and descriptions of the assessed biodiversity and habitat functions. These functions contribute to the overall biodiversity of an ecosystem

Biodiversity and habitat function	Description
Maintenance of threatened species	Maintains populations of threatened or protected species, provides habitat or food for threatened or protected species
Maintenance of high species biodiversity	Habitats that create suitable conditions for diverse species communities and thus support high species biodiversity. Maintains high species diversity (e.g. high Shannon–Wiener index)
Habitat provision	Complex seafloor structures, formed by both biotic and abiotic components, create diverse habitats that support high benthic diversity and biomass (Sokolowski et al. 2015). Three-dimensional features offer shelter and surfaces for attachment, enhancing species richness and ecological function
Genetic variation of habitat-forming species	Habitat-forming species own large genetic variation which ensures potential for adaptation in changing environments and increases resilience of the habitat. Genetic variation reflects evolutionary potential and resilience of the populations of the habitat-forming species, and it is of high importance as it affects an organism’s capability to adapt to environmental change
Fish spawning, nursery grounds and adult feeding areas	Fish have an important role in the ecosystem as predators as well as prey, and by linking the lower trophic levels to the higher ones. The brackish water of the Baltic Sea creates a unique mixture of species that are categorized into pelagic, coastal and demersal species. However, each fish species may utilize different habitats, depending on the life cycle stage and due to their mobile way of life
Provision of bird feeding areas	The Baltic Sea is an important breeding, wintering and feeding area for several species of seabirds. The major groups include ducks, seagulls, terns, auks and waders. Of these, the most conspicuous are the seaducks. The Baltic Sea supports a population of almost 3 million wintering seaducks, making it globally among the most important wintering areas (Bellebaum et al. 2012). Seaduck feeding areas mainly include offshore banks and coastal waters in the outer archipelago (e.g., Nilsson et al. 2016)

### Assessment methodology

To assess the contribution of the different habitat types to ecosystem and biodiversity functions, we used a combination of expert assessments, data, and literature. The relative importance of a habitat type to a function was estimated at a scale from 0 to 3 (3 representing the highest contribution and 0 negligible contribution) as done in Potts et al. (2014). To present an overview of biodiversity components and ecosystem functions provided by different marine habitat types, we produced a matrix following the study by Potts et al. (2014). The scoring of ecosystem functions was conducted at the per-unit-area level (i.e., relative contribution per habitat type), with the aim of identifying which habitat types are particularly significant for sustaining biodiversity and ecosystem functions, regardless of their total areal extent.

First, an initial scoring was carried out in a workshop with a group of experts in marine ecology to assess the relative importance of the habitat types to each biodiversity and ecosystem function. Then a group of 13 experts (including all authors of this article, see Supplementary Information Table S1) with research backgrounds spanning different habitats were invited to assess the functions linked to each habitat type and to conduct a literature review on these linkages. Each expert was assigned habitat types that were closest to their field of expertise (Supplementary Information Table S1).

To support this assessment, targeted literature reviews were conducted by the experts using Scopus and Web of Science, combining habitat type names with ecosystem function terms (e.g., ‘Zostera marina’ AND ‘primary production’). While the search aimed to be comprehensive, it was not a formal systematic review. Peer-reviewed studies from the Baltic Sea were prioritized, and expert knowledge was used to complement gaps in the literature, particularly for unpublished or region-specific observations. This combined approach allowed the experts to synthesize current evidence and refine the scoring of relative importance for each habitat type across multiple functions. The experts were instructed to use both their own knowledge on the topic and to consult external experts as needed (for details of experts consulted see Supplementary Information Tables S2, S3).

In addition, to understand the contribution of habitat types to the maintenance of threatened species, we used the Finnish Inventory Programme for Underwater Marine Diversity data (Forsblom et al. 2024) from years 2007 to 2015 to assess the link between habitat types and threatened macrophyte species (latest red list in Hyvärinen et al. (2019); Supplementary Information S4). After the literature search and data review, the scores were reviewed by the expert group to ensure consistency in the scoring and final scoring were given in a workshop attended by the entire review group. We further assessed confidence of the evidence related to each function with a scale from 1 to 3, where 3 refers to highest confidence with scientific literature from the Northern Baltic Sea, 2 to intermediate confidence with literature from other parts of the Baltic Sea or from freshwater system in the northern Baltic Sea catchment area and 1 to expert opinion.

## Results

**Fig. 1:**
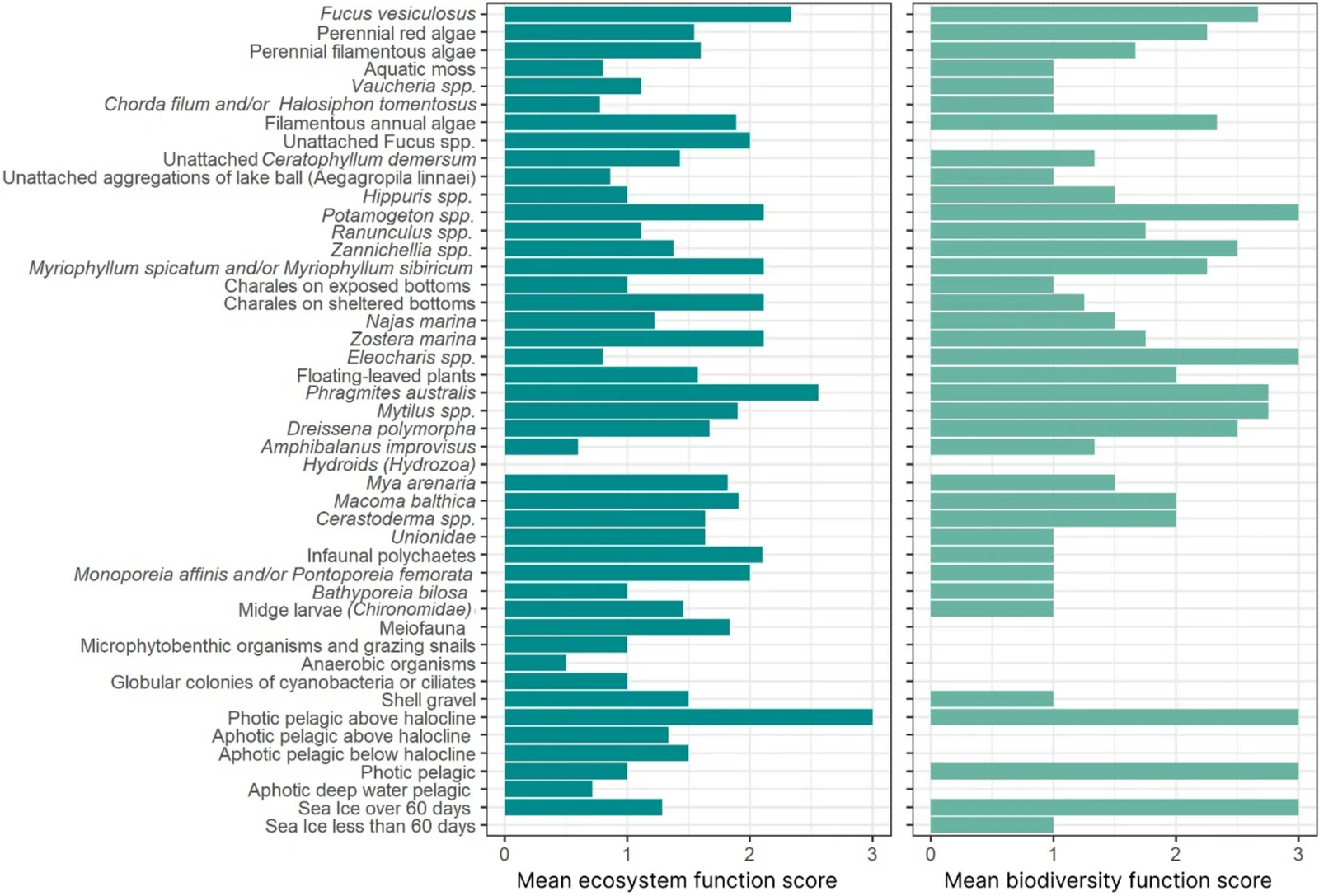
Mean scores for ecosystem and biodiversity functions in the assessed benthic and pelagic habitat types. Score 3 denotes highest contribution and 0 lowest contribution. For full results see Table 3 and Supplementary Information

We assessed the contribution of 40 benthic and 9 pelagic habitat types to marine biodiversity and ecosystem functions in the northern Baltic Sea (Fig. 1), identifying 750 habitat type-function linkages. Of these, 35% were supported by Baltic Sea scientific literature and 29% by expert assessment, while approximately one-third remained unassessed due to data gaps (Table 3).

**Table 3:**
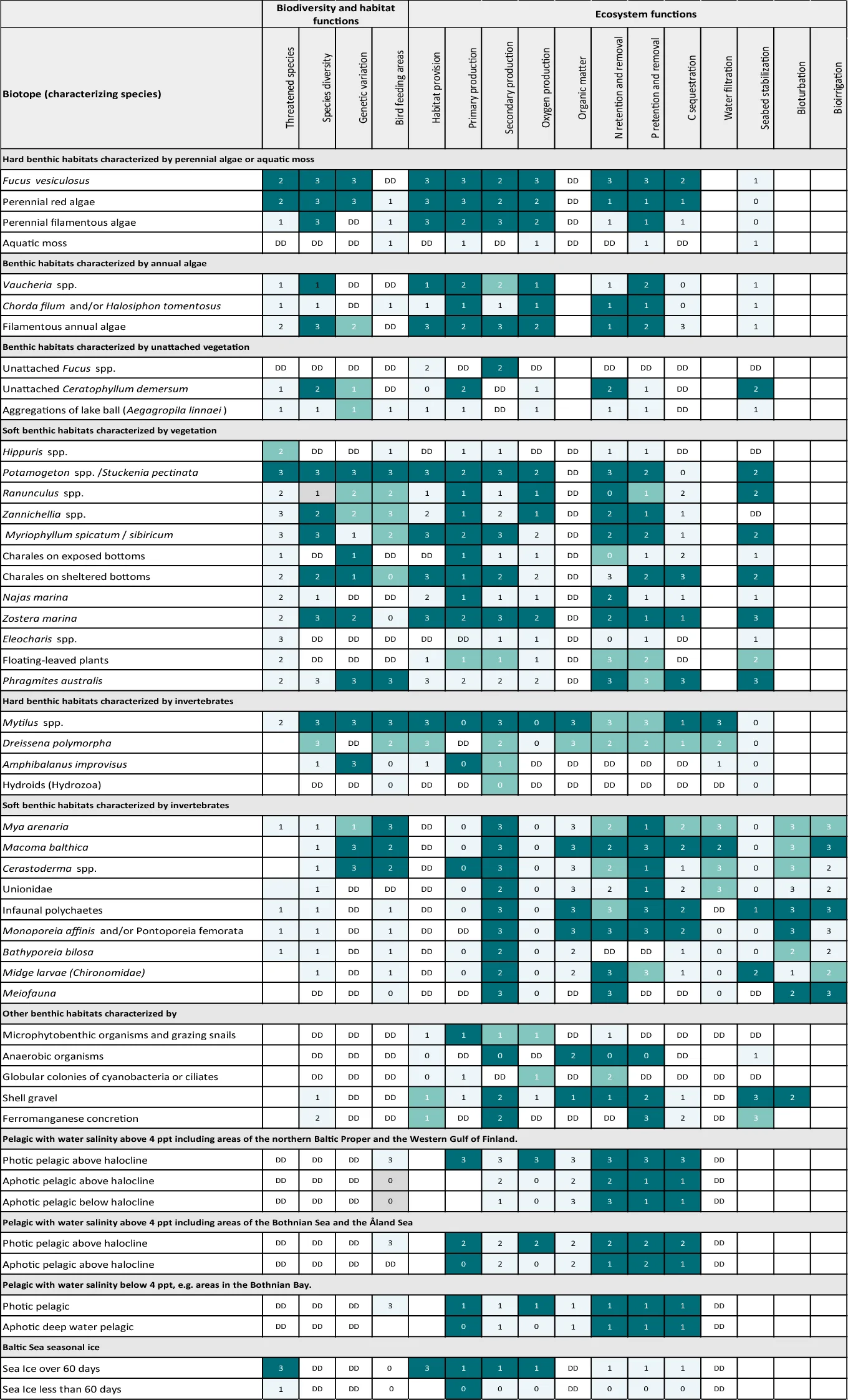
Summary of the assessment results on biodiversity components and ecosystem functions related to underwater habitat types present along the Finnish coast. Numbers in the cells denote the relative contribution of the habitat types to ecosystem function with 3 = high contribution, 2 = moderate contribution, 1 = low contribution, 0 = no contribution, DD = data deficient (not assessed). The shading in the cells presents confidence of the evidence found indication of darker colour referring to higher confidence (dark petrol = Peer-reviewed literature from the northern Baltic Sea, medium green = Peer-reviewed literature from the southern Baltic Sea or from freshwater ecosystem in the drainage area of the northern Baltic Sea, light blue = expert assessment, white = no literature found). Empty cells denote a negligible effect of the habitat type (no linkage). Full results with additional references and rationale are presented in Supplementary Information Table S2

Habitat types dominated by macrovegetation scored highest overall. Soft bottoms with *Potamogeton* and/or *Stuckenia* species received the top scores, followed by habitats with *Z. marina*, hard benthic habitats characterized by *Fucus spp*. and red algae, and soft benthic habitats with Charales (Table 3). Hard benthic habitats dominated by *Mytilus trossulus* matched the functional scores of *Z. marina* habitats, and soft bottoms with *Macoma balthica* also ranked high. For fish, estuaries, benthic unvegetated stone-gravel-sand habitats, and large shallow inlets and bays were identified as the most important habitat types.

The ten most studied benthic habitat types comprised approximately 40% of the available scientific information (Table 3). Benthic habitats characterized by *M. trossulus*, *Fucus* spp., red algae or by perennial filamentous algae as well as soft bottoms characterized by *Z. marina*, *Potamogeton/Stuckenia* spp*.*, *Myriophyllum* spp. or by *M. balthica* were among the most studied habitat types. Most studies on the pelagic habitats concentrated on ecosystem functioning, while only few studies addressed their biodiversity. There were major data gaps in several benthic habitat types (Fig. 1, Table 3). Below, we provide a summary of the key biodiversity and ecosystem functions related to the assessed habitat types (references and quantitative estimates, in Supplementary Information S2).

### Ecosystem functions

#### Net primary production and oxygen production

Hard-bottom habitats with *F. vesiculosus* and pelagic photic zones stand out as key habitat types for net primary and oxygen production (Table 3). Shallow *F. vesiculosus* habitats are highly net autotrophic for most of the year, with annual net ecosystem metabolism up to six times greater than local phytoplankton production, potentially contributing two-thirds of total gross primary production in waters ≤ 5 m (Attard et al. 2019). For vascular plant habitats, productivity varies by species. For instance, *Potamogeton perfoliatus* habitats show higher net primary production (0.77 ± 0.18 mmol O_2_ g^−1^ dwt day^−1^) compared to *Z. marina* (0.39 ± 0.05 mmol O_2_ g^−1^ dwt day^−1^) (Gustafsson and Norkko 2016).

Pelagic photic zones are major productivity hotspots, their output strongly influenced by seasonal light and nutrient conditions. Eutrophication further boosts primary production, particularly during spring blooms. Even in winter, sea ice hosts active autotrophic and heterotrophic communities, supporting primary production in low-light conditions (Tedesco et al. 2017).

Other benthic habitats also contribute to primary production. Photosynthetic cyanobacteria–ciliate mats (Sand-Jensen et al. 1997) and fast-growing epilithic diatoms become seasonally important, especially with longer ice-free periods in the north (Snoeijs 1990). Microphytobenthos has been observed on photic shell gravel beds (Tait et al. 2015), though data are lacking for most habitat types.

#### Secondary production

Secondary production in situ is seldom quantified, with studies focusing on the biomass or abundance of benthic fauna associated with different habitats. A study comparing annual secondary production in situ among bare sedimentary seafloor, stands of mixed vascular plants, habitats characterized *by Z. marina*, *Fucus spp.* and *M. trossulus* showed that mussel reefs had the highest annual secondary production (ranging from 493.4 g Cm^−2^ y^−1^) which was almost double the second highest production measured in the seagrass habitat (278.5 g C m^−2^ y^−1^; Rodil et al. 2020, Table 1).

While data on secondary production rates of soft-bottom macrofauna communities are scarce, estimates of macrofaunal biomasses are available (Table S1). Soft benthic habitats characterized by invertebrates sustain high biomasses of invertebrates with standing stocks of up to > 300 g ww m^−2^ in coastal areas of the Northern Baltic Sea (Gogina et al. 2016); Table 2), depending on the characterizing species communities. Highest biomasses are found in bivalve and polychaete dominated communities (Gusev and Jurgens-Markina 2012).

#### Organic matter decomposition and biodeposition

Benthic fauna on soft and rocky bottoms play a key role in decomposing sedimented organic matter (Table 3), with variation in the efficiency of organic matter decomposition between dominant faunal species and environmental setting (Ehrnsten et al. 2019; Törnroos et al. 2015). For example, the amphipods *M. affinis* and *P. femorata* incorporated approximately 10% of carbon and nitrogen from settled phytodetritus (Karlson et al. 2010). In addition, multispecies communities were more efficient in incorporating carbon and nitrogen from phytodetritus compared to what would have been expected based on single species experiments.

Organic matter processed by filter feeders on hard bottoms can be estimated by measures of biodeposition. Blue mussels show seasonal differences in biodeposition rates varying from 0.52 to 9.76 g dry weight deposited m^−2^ d^−1^, with the highest rates in late autumn (Kautsky and Evans 1987). Zebra mussels have been found to biodeposit approximately one quarter of the suspended particles passing the mussel bed (Daunys et al. 2006). Shell gravel beds formed by zebra mussel shells trap suspended material and accumulate organic matter that is consumed by the deposit feeding fauna associated with the beds (Zaiko et al. 2010). Decomposition of organic matter also occurs on anoxic bottoms by microbial communities, although the rates are lower than in oxic conditions (Noffke et al. 2016).

#### Nitrogen and phosphorus retention and removal

Habitat types with perennial vegetation, soft sediments with invertebrates, and hard substrates with mussels received the highest scores for nitrogen (N) and phosphorus (P) retention and removal. *Fucus, Potamogeton*, *Charales*, and *P. australis* habitat types are especially effective, as perennial macroalgae retain nutrients in their tissues year-round (Indergaard and Knutsen 1990), unlike seasonal filamentous algae with lower standing biomass (Salovius and Kraufvelin 2004).

Permanent nutrient removal in the Baltic Sea mainly occurs via denitrification and phosphorus burial (Carstensen et al. 2020), enhanced by soft-bottom invertebrates that increase oxygenation and microbial activity (Griffiths et al. 2017). *M. affinis* boosts denitrification through surface mixing (Tuominen et al. 1999), and meiofauna can double denitrification rates (Bonaglia et al. 2014). However, deep-burrowing polychaetes like *Marenzelleria* spp. may reverse N flux through bioirrigation (Bonaglia et al. 2013).

*Mytilus* reefs contribute significantly to nutrient cycling and retention, covering 80–95% of rocky seafloor biomass (Kautsky 1981) and meeting 12% of pelagic N and 22% of P demand (Kautsky and Evans 1987). Soft bottoms with *M. balthica*, infaunal polychaetes, and meiofauna also scored high, particularly for P retention. Ferromanganese concretions, covering ca. 11% of Finnish marine seafloors, store large amounts of P and represent a significant phosphorus sink in the northern Baltic Sea (Kaikkonen et al. 2019; Majamäki et al. 2025).

#### Carbon retention and removal

Research on the carbon retention potential of northern Baltic Sea habitat types has focused on macrophyte dominated habitat types, with *Z. marina* meadows being the best studied in terms of distribution and carbon sequestration potential. The average organic carbon stocks in Finnish eelgrass meadows are ca. 630 g C m^−2^ (cf. 4300 g C m^−2^ in Denmark) (Supplementary Information Table S2). This corresponds to a total organic carbon pool of 6.98 t C ha^−1^ (Röhr et al. 2016), suggesting that Finnish eelgrass habitats are minor carbon sinks compared to fully marine eelgrass meadows. As eelgrass only occurs on exposed sandy bottoms, most of the organic carbon produced in the Finnish meadows is exported and stocks vary depending on sediment characteristics, i.e. dry density, porosity and silt content.

The knowledge base regarding the role of other submerged soft bottom angiosperms such as pondweeds and reed beds as carbon stocks in the northern Baltic Sea is still evolving (Wikström et al. 2025). Shallow sheltered shores with dense vegetation are potential hot spots for carbon sequestration and storage, yet empirical evidence is still scarce (Table 2). Southern Baltic Sea reed beds have been shown to store significant amounts of carbon, average C stocks in the reed belt sediments being 17.4 kg C m^−2^ (Buczko et al. 2022). In the northern Baltic Sea, the best understanding of carbon retention on rocky shores is related to *F. vesiculosus* stands. Attard et al. (2019) showed that in abundant *Fucus* habitats, carbon stock varied seasonally between 195–396 g C m^−2^ yr^−1^. Approximately 0.3 kg C m^−2^ yr^−1^ was estimated to be exported elsewhere either as unattached detritus or as dissolved matter.

#### Water filtration

Water filtration is one of the key functions on mussel dominated rocky sublittoral shores, purifying water from organic and inorganic suspended matter through filter feeding (Attard et al. 2020). With estimated filtration rates of up to 5 m^3^ m^−2^ d^−1^, mussel reefs can filter a volume equivalent to the entire water column above them on a daily basis (Attard et al. 2020) or together they can annually filter a water mass corresponding to the water mass of the entire Baltic Sea (Kautsky and Kaustky 2000). In doing so, they transform, store, remove, and above all, recycle substantial amounts of particles and nutrients in the system. Filtration of water also reduces water turbidity. Kotta and Møhlenberg (2002) estimated that the filtering of *Mytilus* in the Baltic Sea reduces up to 67% d^−1^ of the chlorophyll stock (variation 8–67%), whereas the same estimate for *Dreissena* was 29% d^−1^ (5–29%). This, for its part, may be beneficial for the ecosystem net primary production, by, e.g. extending the growth maximum or expanding the depth distribution of algae (Kautsky and Wallentinus 1980).

#### Seabed stabilization

Vegetated and hard-bottom habitat types play a crucial role in stabilizing seabed sediments in the Baltic Sea. Key contributors include seagrass meadows, submerged macrophytes, emergent vegetation, and seafloor mineral precipitates, all of which reduce sediment resuspension and erosion under varying environmental conditions.

Z. *marina* stabilizes sandy sediments in wave-exposed areas, altering grain size and organic matter content while providing infaunal shelter from hydrodynamic forces (Meysick et al. 2019). In sheltered bays and lagoons, dense beds of submerged macrophytes, such as *Chara* spp*.*, *S. pectinata*, and *Potamogeton* spp*.*, significantly reduce sediment and nutrient resuspension and improve water clarity (Kaitaranta et al. 2013; Austin et al. 2017). Emergent macrophytes like *P. australis* are highly effective at reducing resuspension, and the sediment disturbance is lower within reed stands than in open water (Kaitaranta et al. 2013). Floating-leaved species such as *Nuphar lutea* also trap fine, organic-rich particles and limit phosphorus release from sediments.

Hard-bottom features such as ferromanganese concretions and shell gravel enhance sediment stability by forming solid structures on soft seafloors in areas with strong bottom currents. These substrates resist erosion and provide local protection against seabed disturbance (Kotilainen et al. 2018a; Kaikkonen et al. 2019).

#### Bioturbation and bioirrigation

Biomodification of sediment varies by habitat type based on dominant benthic fauna (Table 2). While the amphipods *M. affinis* and *P. femorata* mainly stir the upper 2 cm of the sediments, polychaetes and bivalves burrow deeper into the sediment (Gogina et al. 2016). Amphipods contribute to the diffusive mixing of sediments, whereas bivalves mainly perform surface deposition and polychaetes both conveyor and reverse conveyor belt transport (Kristensen et al. 2012). Sediment reworking rates may, however, differ even between closely related species (Renz and Forster 2013). In shell gravel beds, the redox potential and oxygen penetration depth increased compared to bare sediments (Zaiko et al. 2010). Bonaglia et al. (2014) suggest that although the role of meiofauna bioturbation in aerating the sediment is restricted to the uppermost millimeters, its effect on the nitrogen cycle, through stimulation of denitrifying bacteria, is important. In addition to sediment reworking, benthic fauna modify sediment biogeochemistry by bioirrigation, i.e., by flushing the burrows.

Similar to the differences between species of *Marenzelleria* in particle reworking, also the bioirrigation rates differ between these species and is also dependent on salinity and sediment type (Renz and Forster 2013). In low-saline muddy sediments, the bioirrigation rates were 4.7–12.5 L m^−2^ day^−1^. Hedman et al. (2011) showed that although *Hediste diversicolor* was a more efficient particle reworker in comparison to *Marenzelleria neglecta*, *M. neglecta* showed higher bioirrigation rates, illustrating the importance of species traits on these functions.

### Biodiversity and habitat functions

#### Maintenance of threatened species

Using the Finnish marine underwater inventory program data, we identified threatened macrophyte species (IUCN status of CR, EN or VU, Supplementary Information S5) associated with 14 marine underwater habitat types (Table S3). We further assessed the contribution of habitat types to maintenance of threatened invertebrate, fish, and bird species (Supplementary Information S3).

Among invertebrates, only *Macroplea pubipennis* showed a clear association to shallow soft shores with stands of *Potamogeton* spp. /*Stuckenia* spp. (Vahtera et al. 2018). For the assessed fish species, all habitat types or habitat complexes maintain some endangered or poorly known fish species. Hard benthic habitats maintain near threatened flounder (*Platichthys flesus*, Borg et al. 2014) and poorly known fish species such as sea stickleback (*Spinachia spinachia)* and black goby (*Gobius niger*), whereas sandy banks are important for whitefish and grayling (Veneranta et al. 2013). *Mytilus* reefs have particular importance for endangered waterfowls *Somateria mollissima, Melanitta fusca and Clangula hyemalis,* while *Fucus* stands are linked to *Aythya fuligula*, *A.marila, Clangula hyemalis* (Öst and Kilpi 1998). Shallow soft bottoms with vascular plants serve as feeding grounds and resting places for several waterfowls such as *A. fuligula*, *A. marila, Fulica atra* and reed beds are important for endangered waterfowl such as *Anas acuta*, *Mareca penelope*, *Spatula querquedula* and *Gallinula.*

#### Habitat provisioning

Macroalgae on rocky shores provide important habitat for many species, with habitats characterized by *F. vesiculosus* considered to form particularly important complex year-around habitats (Schagerström et al. 2014, Table 3). In addition to perennial algae, summertime stands of annual *Cladophora glomerata* in warm shallow water provide important habitat for juveniles of gammarids and isopods (Salovius and Kraufvelin 2004) while later in autumn high densities of these mobile fauna are occupying habitats characterized by *Fucus* spp. (Haavisto and Jormalainen 2014). Furthermore, green alga *C. glomerata* and *Ulva* spp. and larger *F. vesiculosus* and *F. lumbricalis* all decrease fish predation on mesograzers like *Idotea spp.* (Wernberg et al. 2013). Marine angiosperms further provide an important habitat, with particularly *Z. marina* meadows creating complex habitats that support high biodiversity and productivity (Gustafsson and Boström, 2009).

Mussel habitats support high biodiversity by adding physical structure that increases habitat complexity (Koivisto and Westerbom 2010) and surface area for grazers (Attard et al. 2020). They also filter suspended materials, enhancing water quality and algal growth, and enrich rocky bottoms with organic matter through biodeposition, benefiting deposit feeders. Similarly, benthic features like shell gravel, ferromanganese concretions, and ciliate or cyanobacterial colonies likely act as ecosystem engineers by providing recruitment surfaces for invertebrates. However, ‘other benthic habitats’ remain unstudied for invertebrate diversity in the Baltic Sea.

Sea ice also constitutes an important habitat type, hosting diverse microbial communities (Kaikkonen et al. 2020) and serving as a crucial breeding and resting platform for seals (e.g., Baltic ringed seal, *Phoca hispida botnica*) (Harkonen et al. 2008).

#### Maintenance of high species diversity

Mytilus reefs and seagrass meadows, along with other vegetated habitat types are among the most species-rich habitat types in the northern Baltic Sea, supporting diverse invertebrate communities (Table 2). *M. trossulus* reefs host over 50 invertebrate species (Westerbom et al. 2019), provide feeding grounds for fish (Borg et al. 2014; Westerbom et al. 2018) and birds (Öst and Kilpi 1998) and are widely distributed across the region.

Seagrass meadows, including mixed meadows of *Z. marina*, *P. perfoliatus, Zannichellia* spp., and *S. pectinata* support high infaunal and epifaunal diversity on otherwise species-poor soft sediments (Gustafsson and Boström 2009, 2014). Infaunal diversity is highest in *Z. marina* habitats compared to *F. vesiculosus*, other algae, and unvegetated sands (Table 1, Henseler et al. 2019). Algal-dominated hard substrates support rich invertebrate and fish communities, with species composition varying across assemblages: fucoids harbor gammarids and gastropods, *Furcellaria* supports more *M. trossulus*, and annual algae attract Chironomidae (Saarinen et al. 2018).

#### Genetic variation of species characterizing habitat types

Most studies on genetic variation focus on *Fucus* and *Zostera*. For *Zostera*, Yu et al. (2020) highlighted how somatic genetic drift during clonal growth leads to genetically distinct modules, contributing to individuality in clonal species. In *Fucus*, several studies (Jormalainen et al. 2017; Preston et al. 2022) have examined genetic diversity and its ecological implications. While the focus has been on these genera, many other habitat types remain data deficient. However, high scores and confidence were assigned to habitats dominated by perennial algae (*Fucus*, red algae, Kostamo et al. 2012), vegetated soft bottoms (*Potamogeton*, *P. australis*), invertebrate-rich soft bottoms (*M. balthica*, *Cerastoderma glauca*), and hard seafloors with *M. edulis* (Kijewski et al. 2019)*.* Communities in the pelagic environment also show high genetic variability (e.g., Sjöqvist et al. 2015).

#### Fish spawning areas, nursery and adult feeding grounds

The pelagic zone provides an important spawning habitat for sprat that depends on saline water to support the development of its floating roe (Ojaveer and Kalejs 2010) (Table 4). In addition, it provides important feeding habitat for migratory fish in their adult stage, such as salmon and trout (Jutila et al. 2003; Kallio-Nyberg et al. 2017). For grayling and whitefish, benthic stone-gravel-sand habitats form important spawning locations in the Baltic Sea (Veneranta et al. 2013;). Hard benthic habitats characterized by perennial algae also provide important spawning areas for herring (Aneer 1989), whitefish, grayling and smelt (Bergström et al. 2021). Soft benthic habitats characterized by vegetation have great importance in providing shelter for spawning, nursery and feeding grounds for fish species such as perch, pike and pikeperch (Bergström et al. 2021). Habitat complexes such as flads and gloes, contain similar characteristics to soft benthic habitats, and also play a very important role as nursery habitats for a variety of fish species, especially spring-spawning species such as perch, as these habitats provide suitable temperature, vegetation coverage, and food (Kraufvelin et al. 2018; Westerbom et al. 2023). Additionally, estuaries support juvenile fish species as pikeperch, whitefish and smelt (Shpilev et al. 2005; Veneranta et al. 2013).

#### Provision of bird feeding areas

There are approximately 80 seabird species occurring in the Baltic Sea (HELCOM 2023) that differ profoundly in their habitat and food requirements. Seaduck feeding areas mainly include offshore banks and coastal waters in the outer archipelago (Nilsson et al. 2016), feeding on mainly benthic invertebrates on rocky or muddy substrates. Up to 95% of the diet of eider ducks and a significant share in the diet of long-tailed ducks is composed of *M. trossulus* (Öst and Kilpi 1997; Waldeck and Larsson 2013). These numerous birds are therefore completely dependent on the availability of this food resource (Nilsson et al. 2016). Gulls and terns form the second largest group, and their breeding and foraging sites are found in the inner and outer archipelago. Both are piscivores and find their prey from a variety of shallow habitats, spanning from the open pelagic realm to the shallow bottoms in the inner archipelago. Piscivorous auks prefer barren rocky islets as breeding grounds at the fringe to the open sea where they feed on both pelagic and benthic fish. Waders utilize the supralitoral and the geolitoral on muddy, sandy and rocky shorelines, where they prey on mollusks, polychaetes and crustaceans (Table 4).

**Table 4:**
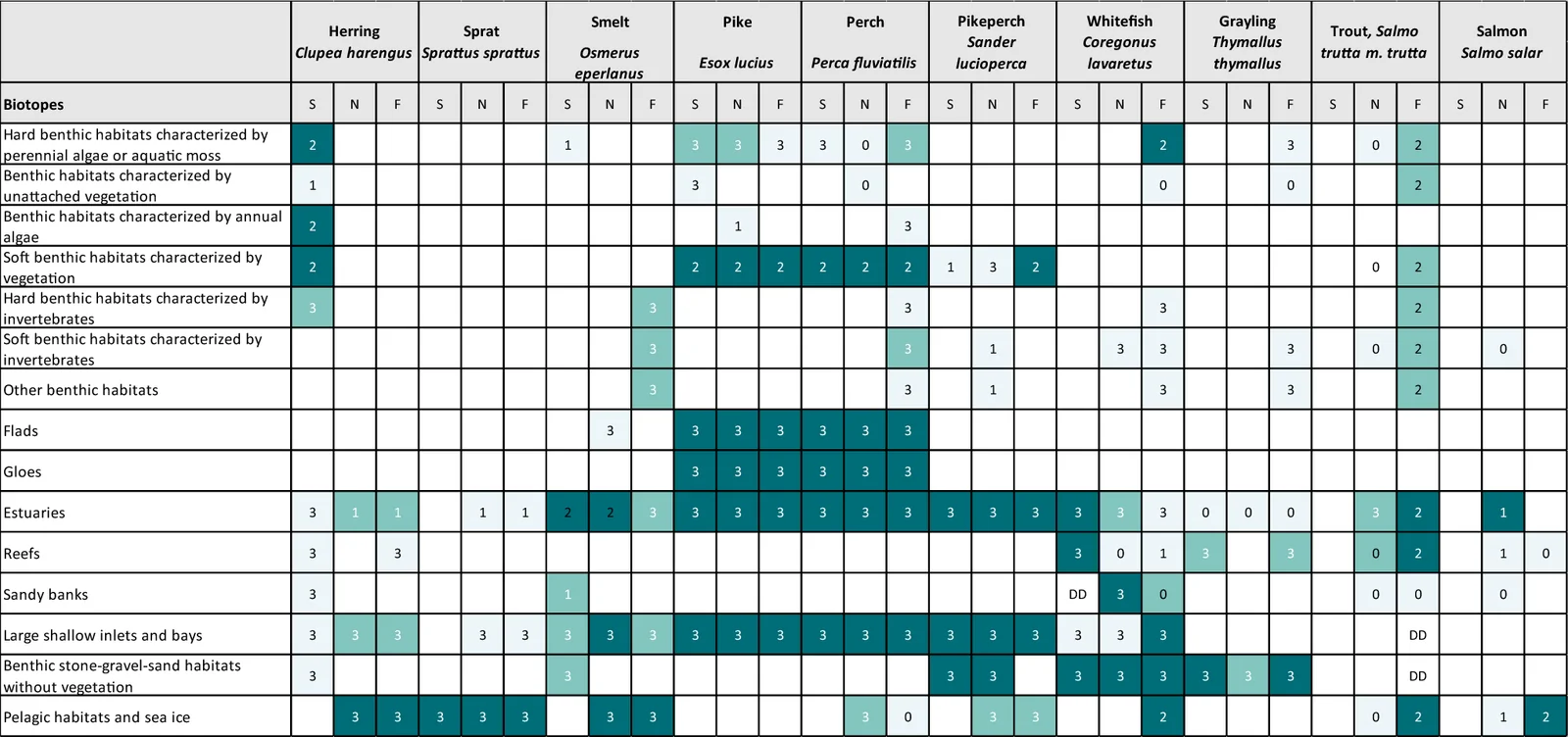
Summary of the assessment results on links between different life stages of selected fish species (S = spawning, N = nursery grounds, F = adult feeding areas) and underwater habitats along the Finnish coast. Numbers in the cells denote the relative contribution of the habitat types to fish with 3 = high contribution, 2 = moderate contribution, 1 = low contribution, 0 = no contribution DD = data deficient (not assessed). The shading in the cells presents confidence of the evidence found indication of darker colour referring to higher confidence (dark petrol = Peer-reviewed literature from the northern Baltic Sea, medium green = Peer-reviewed literature from the southern Baltic Sea or from freshwater ecosystem in the drainage area of the northern Baltic Sea, light blue = expert assessment, white = no literature found. Empty cells denote a negligible effect of the fish species

## Discussion

In this study we assessed contribution of underwater habitat types to different ecosystem and biodiversity functions to identify key habitat types for ecosystem functionality. This was done in our case study area, the northern Baltic Sea, by using scientific literature, data, and expert assessment.

Although we identify key habitats based on functional diversity, our approach designed to capture both the breadth of functions supported by habitats and the distribution and rarity of individual functions across habitat types. Depending on the scale and conservation objective, either approach may be relevant. By providing an assessment of functional roles across habitat types, the study offers a foundation for prioritizing conservation, restoration, and management actions in a way that reflects both multifunctionality and the critical importance of specific habitats for individual functions.

Soft benthic habitats characterized by *Potamogeton* spp./*Stuckenia* spp.*, Myriophyllum* spp., *Z. marina, M. balthica* and *M. affinis* were assessed to be highly important for most functions. Among hard benthic habitats, those characterized by *Fucus* spp. and *M. trossulus* were assessed to have high functionality. In addition, habitats characterized by *P. australis* were considered a key habitat type both in terms of supporting biodiversity and ecosystem functions. Among the above-mentioned habitat types, benthic habitats characterized by *Fucus* spp. and *M. affinis/P. femorata* are classified as endangered (Kotilainen et al. 2018a). For fish, estuaries, benthic unvegetated stone-gravel-sand habitats, and large shallow inlets and bays were the most important habitats.

Different habitat types are important for different functions, and it is important to note that in this assessment, key habitat types are defined as habitat types ranking highest based on how strongly they contribute to the functions assessed in this study. However, in a biogeographical context, many habitat types, such as those found in offshore areas, may proportionally contribute to significant functions due to their large areas, although they may not emerge as key habitats per unit area when compared to other underwater habitat types (Borja et al. 2016; Virtanen et al. 2024). Our results thus highlight both the limited understanding of the functional roles of several habitat types and a clear research bias toward more studied habitats, emphasizing the need for broader, spatially inclusive assessments to fully capture the contributions of all habitats to ecosystem functioning.

### Knowledge gaps regarding functions and habitat types

Despite extensive research on certain ecosystem functions and habitat types, significant knowledge gaps remain. For instance, coastal carbon sequestration and burial processes are still only partly understood, particularly regarding the amount of carbon stored in different habitat types and the mechanisms governing carbon cycling (Wikström et al. 2025). This gap hinders assessment of the Baltic Sea’s role as a carbon sink and limits predictions of how functions may shift with increasing habitat degradation and climate change (Reckermann et al. 2022). Similarly, denitrification, a key process in nitrogen removal, is another identified knowledge gap. While some studies have examined denitrification rates, little is known about how specific benthic communities influence this process (Bonaglia et al. 2014; Wikström et al. 2025). Understanding the role of marine habitat types in nitrogen cycling as well as the seasonal dynamics in nitrification/denitrification is essential for developing effective nutrient management strategies.

In comparison to ecosystem functions, the contribution of habitat types to biodiversity functions was more uncertain both in terms of the number of data deficient habitat types and literature evidence from the northern Baltic Sea region, including identified knowledge gaps on habitat provision of habitat types to overall biodiversity and threatened species. This is likely partly caused by the inherent challenges of comparing different types of functions, as they represent distinct ecological processes. Many ecosystem functions such as carbon retention can be quantified using standardized metrics, whereas many biodiversity functions, like habitat provision or support for biodiversity, are more qualitative and context-dependent, i.e., varying depending on both ecological and spatial settings. Nevertheless, all these aspects contribute to overall ecosystem functioning and the delivery of ecosystem services. This highlights the need for a better understanding of the ecosystem structures and functions that underpin marine ecosystem service provisioning (Inacio et al. 2020; Jernberg et al. 2024). Many of these relationships remain poorly understood, raising the risk of over or underestimating the value of certain habitat types.

The findings of this study highlight that the knowledge base of underwater habitat types in the northern Baltic Sea remains incomplete, posing challenges for effective conservation and management. The focus on a limited number of well-studied habitat types has resulted in a poor understanding of many others, which are consequently often assumed to have geographically restricted distributions. This has been the case, for example, with FeMn concretion fields, which were previously overlooked from an ecological perspective but are now known to occur abundantly in the northern Baltic Sea (Kaikkonen et al. 2019). The lack of attention to such habitats not only hampers our understanding of the region’s ecological dynamics but also increases the likelihood that they will be excluded from conservation priorities. Consequently, these knowledge gaps may lead to management decisions that unintentionally compromise broader ecosystem functioning.

### Addressing uncertainties in the assessment

Identification of key habitat types based on ecosystem functions often relies on limited data, introducing significant uncertainties. Extrapolating findings from single studies or locations to broader regional scales can lead to misinterpretations of the significance and function of different habitat types. In this study, the use of expert knowledge was therefore central to the assessment. Experts were given the lead in evaluating each habitat type because they were best positioned to scrutinize the relevance and applicability of the available evidence to the northern Baltic Sea context. Expert input was essential to complement published data, particularly in cases where studies were limited, highly localized, or context specific. This combined approach allowed the experts to refine and validate the scoring of relative importance for each habitat type across multiple ecosystem functions, ensuring a robust, evidence-based assessment, while explicitly acknowledging the inherent uncertainties in the underlying data. While this approach aids in understanding the differences between habitat types and their role in the ecosystem for management purposes, we acknowledge the fact that this is a simplification of complex ecosystem processes. For example, differences in species biomasses and related functions caused by various environmental conditions are not captured (Wohlgemuth et al. 2017), and this should be kept in mind when using the results.

Classification systems based on functions may not always align with observed ecological patterns, particularly in dynamic environments like the Baltic Sea. Some habitat types provide multiple, overlapping functions, while others vary seasonally or spatially (Attard et al. 2019; Rodil et al. 2021), complicating categorization. Consequently, management strategies based on such classifications must acknowledge uncertainty and remain adaptable to new research. By providing a clearer understanding of the functional roles of different habitats, this framework also lays the groundwork for more accurate ecosystem service assessments, where linking habitat types to the services they support is critical despite often incomplete data.

### Identifying key habitats is essential for management actions

Understanding which habitat types are most important for sustaining biodiversity and ecosystem functions is essential for evaluating how human activities and environmental change affect coastal ecosystems (e.g., Barbier et al. 2011). Key habitats such as seagrass meadows, perennial macroalgal beds, and charophyte stands support nutrient retention, carbon storage, and essential fish habitats, but these functions are vulnerable to pressures, such as eutrophication and construction activities (Korpinen et al. 2012). Future conservation efforts can benefit from the data collected in this study by incorporating it into spatial prioritization analyses (Virtanen et al. 2018), which help identify key areas for protection based on ecological significance. Additionally, identifying the specific ecosystem functions and biodiversity components most at risk can guide targeted conservation and restoration efforts. Recognizing habitat types that act as functional hotspots makes it possible to spatially target conservation and management actions where they will have the greatest ecological impact (Kuismanen et al. 2023).

Monitoring the condition of these functionally important habitats can provide early warning signals of degradation, while also helping to link pressures directly to ecosystem processes and services. This approach is particularly relevant in the context of international commitments, such as the HELCOM Baltic Sea Action Plan, the EU Biodiversity Strategy, and emerging blue carbon initiatives, which emphasize safeguarding habitats that contribute both to biodiversity and to climate mitigation. By providing a basis for ecosystem accounting and integrating habitat values into policy frameworks, habitat-type-based functional assessments not only clarify ecological priorities but also directly support conservation planning, restoration, and ecosystem-based management in the northern Baltic Sea.

The method in this assessment is relatively simple and thus similar assessments could be conducted in other marine areas with the local experts and literature. Based on our experience, key issues for a successful assessment include choosing a habitat classification system that serves for the purpose of the study and including a group of professionals that already have good expertise on the subject (and using additional experts if it seems that more expertise is needed). This kind of review may be very laborious, particularly in areas where the number of habitats is much higher than the Baltic Sea. Thus, both the chosen classification system and good experts may ease the task and help acknowledging key habitats, which can improve marine management and conservation, and potentially highlight the role of some habitats that have not traditionally been considered so important for the ecosystem functioning.

Our study focused on the relative functional importance of habitat types per unit area to identify those that are especially critical for multiple ecosystem functions. We acknowledge, however, that ecosystem-scale contributions also depend on spatial extent and on the rarity or redundancy of individual functions across habitats. Habitats supporting only one or a few functions may therefore be highly important at landscape or regional scales if those functions are weakly represented elsewhere. Spatial extent of underwater marine habitats has already been carried out for Finnish marine areas (Virtanen et al. 2024), and combining these data with per-unit-area functional role of each habitat would be a valuable next step. By highlighting functionally important habitats and potential hotspots of multiple ecosystem services, our findings provide a practical basis for management, supporting monitoring, marine spatial planning, and the designation of protected areas. Where habitats providing specific functions have been degraded, targeted restoration, such as seagrass transplantation or charophyte re-establishment, can maximize returns for both biodiversity and functioning. Considering functional importance with habitat extent also enables incorporation into ecosystem accounting, directly linking ecological value to policy and decision-making. Importantly, management decisions should not rely solely on functional diversity, as habitats supporting only a few functions may still play a crucial role at broader spatial scales. Altogether, this approach helps translate scientific understanding into actionable guidance for conservation and ecosystem-based management in the northern Baltic Sea.

## Supplementary Information

Below is the link to the electronic supplementary material.

Supplementary file1 (XLSX 119 KB)
