# Publisher Correction: Key underwater habitat types of the northern Baltic Sea: An assessment of biodiversity and ecosystem functions

**DOI:** 10.1007/s13280-026-02440-9

**Published:** 2026-06-25

**Authors:** Laura Kaikkonen, Susanna Jernberg, Fiia Haavisto, Christoffer Boström, Camilla Gustafsson, Suvi Kiviluoto, Kirsi Kostamo, Sanna Kuningas, Harri Kuosa, Essi Lakso, Annica Långnabba, Henrik Nygård, Mats Westerbom

**Affiliations:** 1 Finnish Environment Institute Syke, Latokartanonkaari 11, 00790 Helsinki, Finland; 2 University of Helsinki, Helsinki, Finland; 3 Metsähallitus, Parks and Wildlife Finland, Opastinsilta 12 C, 00521 Helsinki, Finland; 4 Environmental and Marine Biology, Åbo Akademi University, Henriksgatan 2, 20500 Turku, Finland; 5 Tvärminne Zoological Station, University of Helsinki, J. A. Palméns Väg 260, 10900 Hanko, Finland; 6 Natural Resources Institute Finland (Luke), Latokartanonkaari 9, 00790 Helsinki, Finland; 7 Natural Resources Institute Finland (Luke), Wolffintie 35, 65200 Vaasa, Finland

**Publisher Correction to: Ambio** **https://doi.org/10.1007/s13280-026-02391-1**

In this article, two versions of Table 4 (with and without colour) were processed mistakenly during the production process. The correct version of Table 4 is provided in this correction.

**Table 4:**
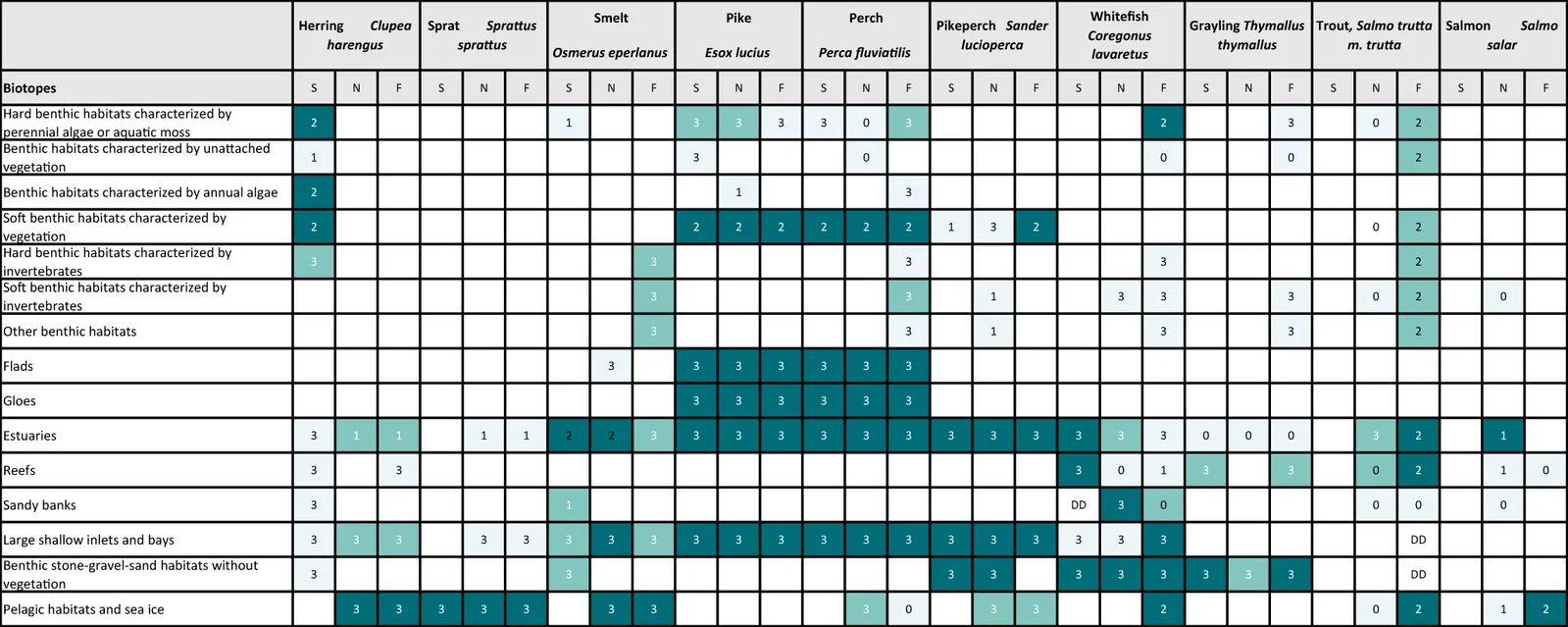
Summary of the assessment results on links between different life stages of selected fish species (S = spawning, N = nursery grounds, F = adult feeding areas) and underwater habitats along the Finnish coast. Numbers in the cells denote the relative contribution of the habitat types to fish with 3 = high contribution, 2 = moderate contribution, 1 = low contribution, 0 = no contribution DD = data deficient (not assessed). The shading in the cells presents confidence of the evidence found indication of darker colour referring to higher confidence (dark petrol = Peerreviewed literature from the northern Baltic Sea, medium green = Peer-reviewed literature from the southern Baltic Sea or from freshwater ecosystem in the drainage area of the northern Baltic Sea, light blue = expert assessment, white = no literature found. Empty cells denote a negligible effect of the fish species

The original article has been corrected.

